# Evaluating the predictive performance of presence–absence models: Why can the same model appear excellent or poor?

**DOI:** 10.1002/ece3.10784

**Published:** 2023-12-18

**Authors:** Nerea Abrego, Otso Ovaskainen

**Affiliations:** ^1^ Department of Biological and Environmental Science University of Jyväskylä Jyväskylä Finland; ^2^ Department of Agricultural Sciences University of Helsinki Helsinki Finland; ^3^ Department of Biology, Centre for Biodiversity Dynamics Norwegian University of Science and Technology Trondheim Norway; ^4^ Organismal and Evolutionary Biology Research Programme, Faculty of Biological and Environmental Sciences University of Helsinki Helsinki Finland

**Keywords:** accuracy, AUC, cross‐validation, discrimination, joint species distribution model, max‐kappa, max‐TSS, presence–absence model, sensitivity, specificity, Tjur's *R* ^2^

## Abstract

When comparing multiple models of species distribution, models yielding higher predictive performance are clearly to be favored. A more difficult question is how to decide whether even the best model is “good enough”. Here, we clarify key choices and metrics related to evaluating the predictive performance of presence–absence models. We use a hierarchical case study to evaluate how four metrics of predictive performance (AUC, Tjur's *R* ^2^, max‐Kappa, and max‐TSS) relate to each other, the random and fixed effects parts of the model, the spatial scale at which predictive performance is measured, and the cross‐validation strategy chosen. We demonstrate that the very same metric can achieve different values for the very same model, even when similar cross‐validation strategies are followed, depending on the spatial scale at which predictive performance is measured. Among metrics, Tjur's *R* ^2^ and max‐Kappa generally increase with species' prevalence, whereas AUC and max‐TSS are largely independent of prevalence. Thus, Tjur's *R* ^2^ and max‐Kappa often reach lower values when measured at the smallest scales considered in the study, while AUC and max‐TSS reaching similar values across the different spatial levels included in the study. However, they provide complementary insights on predictive performance. The very same model may appear excellent or poor not only due to the applied metric, but also how predictive performance is exactly calculated, calling for great caution on the interpretation of predictive performance. The most comprehensive evaluation of predictive performance can be obtained by evaluating predictive performance through the combination of measures providing complementary insights. Instead of following simple rules of thumb or focusing on absolute values, we recommend comparing the achieved predictive performance to the researcher's own a priori expectations on how easy it is to make predictions related to the same question that the model is used for.

## Graphical abstract

We demonstrate that the very same metric can achieve different values for the very same model, even when similar cross‐validation strategies are followed, depending on the spatial scale at which predictive performance is measured. Tjur's *R* ^2^ and max‐Kappa often reach lower values when measured at the smallest scales considered in the study, while AUC and max‐TSS reaching similar values across the different spatial levels included in the study. However, we show that a low value of Tjur's *R* ^2^ or max‐Kappa should not uncritically be taken as evidence for poor predictive performance, neither should a high value of AUC or max‐TSS be accepted as proof of high predictive performance.

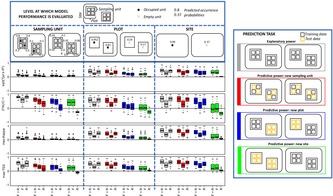

## 1 INTRODUCTION

In species distribution modeling, a model's predictive performance is routinely assessed by determining how well the model is able to predict either the same data that it was originally fitted to (explanatory power) or to predict validation data independent from the training data used for model fitting (predictive power on hold‐out data). How exactly to partition the full data into training and validation data, and what method to use to evaluate the model's predictive performance, has been the focus of a large body of literature (e.g., Allouche et al., 2006; Araújo et al., 2005; Elith et al., 2006; Hijmans, 2012; Liu et al., 2011; Norberg et al., 2019; Vaughan & Ormerod, 2005). When comparing multiple models, a model yielding higher predictive performance is clearly to be favored over a model yielding lower predictive performance, yet a more difficult question is how to decide if even the best model is “good enough”. To facilitate the interpretation of model evaluation results, simple rules of thumb have been proposed to classify predictive performances into categories such as “excellent,” “good,” or “poor” (Araújo et al., 2005; Guisan et al., 2017; Swets, 1988). However, as we argue in this article, following such simplified rules of thumb can lead to misleading interpretations, because the level of predictive performance achieved will simultaneously depend on multiple factors that should explicitly be accounted for.

The level of predictive performance achieved depends not only on how optimally the model has been chosen, but also on the nature of the study system and on the study design—especially on the range and representation of environmental conditions present in the data (e.g., Guillera‐Arroita et al., 2015; Jiménez‐Valverde et al., 2013; Peterson et al., 2008; Segurado & Araújo, 2004). Furthermore, the very same model can achieve very different levels of predictive performance depending on the spatial and/or temporal scale at which the prediction task is performed (Aguirre‐Gutiérrez et al., 2013; Sofaer et al., 2019; Termansen et al., 2006; Wisz et al., 2008), and how the sampling units are partitioned into training and validation data (e.g., Araújo et al., 2005; Bahn & McGill, 2013; Roberts et al., 2017). Thus, when considering how good a model is, it is not sufficient to merely consider the absolute value of a particular metric of predictive performance. Rather, it is also necessary to account for the many factors that influence the values achieved. Many ecologists, whether conducting their own work or evaluating that of others, may not be sufficiently aware of the complexities related to evaluating predictive performance, which motivated the present article.

Predictive performance can be measured in terms of, for example, accuracy, discrimination, precision, or calibration (Norberg et al., 2019). Here we focus on measures discrimination, that is, measures of how well a model discriminates between sampling units where a focal species is present and sampling units from which it is absent (Allouche et al., 2006; Jiménez‐Valverde, 2012; Norberg et al., 2019; Tjur, 2009). While there are also many other metrics for measuring predictive performance in species distribution modeling (Guisan et al., 2017; Sofaer et al., 2019), here we focus on the four metrics of AUC, Tjur's *R* ^2^, max‐Kappa, and max‐TSS. The abbreviation AUC stands for the “area under the receiver operating characteristic curve,” and this metric was initially developed in the fields of signal processing and medical science (Hanley & McNeil, 1982). It was proposed as a measure of predictive performance for SDMs by Fielding and Bell (1997) and has become the most commonly applied metric for evaluating the predictive performance of presence–absence SDMs (Elith et al., 2006; Liu et al., 2011; Pearce & Ferrier, 2000). However, it has also been subject to criticism, because, for example, it can reach a high value if including a large number of sites where the species is very unlikely to occur (Lobo et al., 2008; Peterson et al., 2008; see also Sofaer et al., 2019, for a variant that circumvents this problem). A more recent metric for measuring predictive performance in presence–absence models is Tjur's coefficient of discrimination (Tjur, 2009), henceforth Tjur's *R* ^2^. Tjur's *R* ^2^ was developed as an alternative to other coefficients of determination for logistic regression models (Tjur, 2009) and introduced in the SDM literature by Ovaskainen et al. (2016). One advantage of Tjur's *R* ^2^ is its resemblance to the *R* ^2^ of the linear model, allowing its intuitive interpretation as the proportion of variance explained by the model. While Tjur's *R* ^2^ is still less commonly used than AUC, it has recently gained increasing popularity as a measure of predictive performance in SDM (e.g., Kotta et al., 2019; Mang et al., 2018; Tikhonov, Duan, et al., 2020; Tikhonov, Opedal, et al., 2020; Zhang et al., 2018). The metrics Kappa and TSS are based on threshold probabilities above which a species is considered to be present and below which is considered absent. These predicted presences and absences are then contrasted to the observed presences and absences through the so‐called 2 × 2 confusion matrix. Kappa evaluates the overall accuracy of model predictions, corrected by the accuracy expected to occur by chance (Allouche et al., 2006; Shao & Halpin, 1995). Allouche et al. (2006) criticized Kappa due to its tendency to evaluate predictive performance more positively for common than for rare species and proposed the true skill statistics (TSS) as an alternative for Kappa. Also the metric AUC has been favored because it is relatively insensitive to the prevalence of the focal species (Franklin et al., 2009; Manel et al., 2001; McPherson et al., 2004). Both Kappa and TSS are based on a user‐selected probability threshold, which can be difficult to use in an informed way and makes comparison among studies difficult. As a generic solution, Guisan et al. (2017) proposed to try out all probability thresholds and apply the ones that maximize the metrics, leading to max‐Kappa and max‐TSS. This is somewhat analogous to the definition of AUC based on integrating over all possible probability thresholds (Fielding & Bell, 1997; Manel et al., 2001).

In attempts to provide clear guidelines for how to assess predictive performance, AUC values have been split into various performance classes, with AUC > 0.9 corresponding to “excellent,” 0.8 < AUC < 0.9 to “good,” 0.7 < AUC < 0.8 to “fair,” 0.6 < AUC < 0.7 to “poor,” and 0.5 < AUC < 0.6 to “fail” (Araújo et al., 2005). Such rules of thumb for evaluating predictive performance have been adopted in several studies (e.g., Gogol‐Prokurat, 2011; Marmion et al., 2009; Smolik et al., 2010; Thuiller et al., 2006; Wang et al., 2020). In this article, we argue that using performance classes or focusing on the absolute values of the metrics can result in misleading interpretations and oversimplified conclusions regarding predictive performance. In evidence of this claim, we show that the very same model can achieve either a high or a low value also for a single metric of predictive performance, depending on the model evaluation strategy. To advance the *status quo*, we clarify what key choices must be made when evaluating predictive performance. To this aim, we use a hierarchical case study of wood‐inhabiting fungi to describe how AUC, Tjur's *R* ^2^, max‐Kappa, and max‐TSS should be interpreted, how they relate to each other and species prevalence, how a model's predictive performance will vary with the model structure (most importantly, the fixed and random effects included), the spatial scale at which predictive performance is measured, and the cross‐validation strategy chosen.

## 2 MATERIALS AND METHODS

### 2.1 Overall accuracy, sensitivity and specificity

We focus here on four metrics of predictive performance: true skill statistic (max‐TSS), Cohen's Kappa, Tjur's *R* ^2^, and AUC. While all these metrics are used to assess how well the fitted models distinguish between occupied and empty sampling units, they have different mathematical formulations, and thus evaluate model performance from different perspectives. To introduce these measures and discuss their properties, we first introduce some basic notation and terminology. We denote by *y* the data, so that *y*_*i*_ = 1 if the sampling unit *i* is occupied, and *y*_*i*_ = 0 if it is empty. We further denote by $p_{i}$ the model‐predicted occurrence probability for the sampling unit *i*. Some of the measures we consider are based on the idea of thresholding, that is, considering that the model predicts a presence (respectively, absence) if the probability is greater (respectively, smaller) than the selected threshold $t^{*}$. We denote thresholding‐based predictions by $t_{i}$ so that $t_{i}=1$ if $p_{i}\geq t^{*}$, and $t_{i}=0$ if $p_{i}<t^{*}$. With thresholding‐based predictions, it is natural to use the 2 × 2 confusion matrix as a basis of model evaluations. True positives are those datapoints (the number of which we denote by *a*, following the notation of Allouche et al. (2006)) for which the model correctly predicts species presence ($t_{i}=1$ and $y_{i}=1$); true negatives are those *d* datapoints for which the model correctly predicts species absence ($t_{i}=0$ and $y_{i}=0$); false positives are those *b* datapoints for which the model falsely predicts species presence ($t_{i}=1$ but $y_{i}=0$); and false negatives are those *c* datapoints for which the model falsely predicts species absence ($t_{i}=0$ but $y_{i}=1$). Overall accuracy is defined as $\left(a+d\right)/n$, sensitivity as $a/\left(a+c\right)$, and specificity as $d/\left(b+d\right)$, where n=a+b+c+d the total number of datapoints. Thus, overall accuracy measures the proportion of all datapoints (whether actual presences or absences) that the model predicts correctly, sensitivity measures the proportion of actual presences that the model predicts correctly, and specificity measures what proportion of actual absences that the model predicts correctly.

**FIGURE 1:**
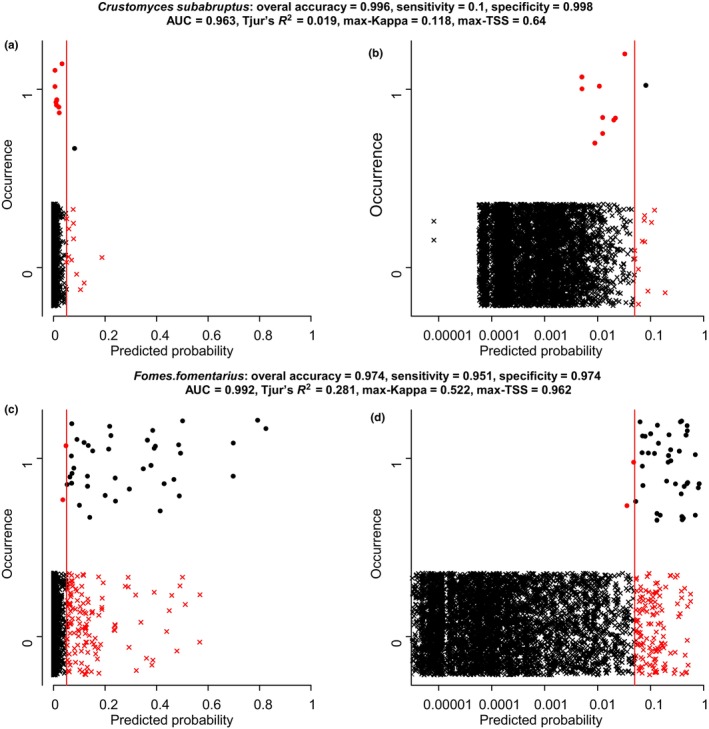
Illustrations of model evaluations based on comparing actual occurrences (*y*‐axis showing presence or absence; note that datapoints jiggered vertically to how overlapping data) by model predictions (*x*‐axis). The panels show the data for the wood‐inhabiting species *Crustomyces subabruptus* (AB) and *Fomes fomentarius* (CD), with probabilities shown either in the linear (AC) or logarithmic (BD) scale. The red vertical lines show a probability threshold set arbitrarily to $t^{*}=0.05$. The black symbols correspond to correct predictions (black dots show true positives and back crosses show true negatives) and the red symbols to incorrect predictions (red dots show false negatives and red crosses show false positives). The values of the model evaluation metrics considered in this paper are shown on top of the panels.

Figure 1 illustrates these concepts by two examples of model predictions versus actual species occurrences. We note in passing that these examples derive from the case study of wood‐inhabiting fungi that we will consider in more detail below, but at this point it is not yet necessary to know in detail how the predictions were generated. Both species that we consider in Figure 1 are rare: the prevalence of *Crustomyces subabruptus* is 0.2% (occurring 10 times out of the 5097 sampling units), and the prevalence of *Fomes fomentarius* is 0.8% (occurring 41 times). As the species are so rare, the models predict very low probabilities for most sampling units, for which reason we have used both the linear and the log‐scale to show the full range of probabilities (Figure 1). As for the interpretation of Tjur's *R* ^2^, it is important to know that the predicted probabilities are well calibrated, we note that this is the case: the sum over all 5097 occurrence probabilities for *C. subabruptus* is 10.7 (which close to the number of 10 occurrences), whereas for *F. fomentarius* it is 41.1 (which is close to the number of 41 occurrences). The model predictions do not reach high occurrence probabilities for any sampling units, and thus selecting, for example, a threshold of $t^{*}=0.9$ would suggest that, for example, *C. subabruptus* would not be present anywhere. For this reason, we have used $t^{*}=0.05$ as the threshold, as illustrated in Figure 1 by the red vertical lines. With this threshold, the overall accuracy is very high for both species (0.995 for *C. subabruptus* and 0.974 for *F. fomentarius*), reflecting the fact that many more sampling units are predicted correctly (black symbols) than incorrectly (red symbols). Even if the low value of the threshold means that we have attempted to increase sensitivity with a potential cost for specificity, the sensitivity in *C. subabruptus* is only 0.1, as only one of the true presences pass the selected threshold. For *F. fomentarius*, sensitivity is high (0.93), as almost all true presences pass the selected threshold. Specificity is very high for both species, reflecting the fact that the vast majority of empty sampling units obtain very low model predictions.

### 2.2 Model evaluation measures: max‐TSS, max‐Kappa, Tjur's *R* ^2^, and AUC

Three out of the four model evaluation measures that we consider here (max‐TSS, max‐Kappa, and AUC) are derived from a thresholding‐based perspective, whereas Tjur's *R* ^2^ is directly based on occurrence probabilities without a reference to thresholding (Table 1). AUC overcomes the difficulty of the user needing to choose a specific threshold by integrating over all possible thresholds, which also leads AUC to have a probabilistic interpretation (Table 1). Kappa and TSS, however, require choosing a specific threshold $t^{*}$. Here we will follow the often‐used alternative for selecting the threshold: max‐Kappa and max‐TSS are defined as the values of Kappa and TSS for the threshold that maximizes their values (Guisan et al., 2017).

**TABLE 1:** The four metrics of model evaluation considered in this paper, their formulations, ranges, and descriptions.

Metric	Formula	[Range] (random expectation)	Description
Area under the operator curve (AUC)	$\frac{\sum_{i,j}1_{p_{i}>p_{j}}y_{i}\left(1-y_{j}\right)\;}{\sum_{i,j}y_{i}\left(1-y_{j}\right)\;},$ where the indicator function $1_{p_{i}>p_{j}}$ obtains the value of one if $p_{i}>p_{j}$, whereas otherwise it obtains the value of zero	[0…1] (0.5)	The proportion of cases for which the occurrence probability for a randomly chosen occupied sampling unit is higher than the occurrence probability for a randomly chosen empty sampling unit. Can be equivalently defined as the integral of the receiver operating characteristic (ROC) curve, plotting sensitivity against 1—specificity over all thresholds (Hanley & McNeil, 1982)
Tjur's *R* ^2^	${\bar{p}}_{y=1}-{\bar{p}}_{y=0}$ $=\frac{\sum_{i}y_{i}p_{i}}{\sum_{i}y_{i}}-\frac{\sum_{i}\left(1-y_{i}\right)p_{i}}{\sum_{i}\left(1-y_{i}\right)}$	[−1…1] (0)	The difference in the average occupancy probabilities between occupied and empty sampling units (Tjur, 2009). Can be also interpreted as variance explained by the binary model, hence being to some extent equivalent of the usual *R* ^2^ of linear models (Tjur, 2009)
True skill statistic (TSS)	Sensitivity + specificity – 1	[−1…1] (0)	TSS compares the number of correct predictions, minus those attributable to random guessing, to that of a hypothetical set of perfect predictions (Allouche et al., 2006)
Cohen's Kappa	$\frac{A_{o}-A_{e}}{1-A_{e}},$ where $A_{o}$ and $A_{e}$ are, respectively, the observed and expected total accuracy	[−1…1] (0)	Overall accuracy of model predictions, corrected by the accuracy expected to occur by chance (Allouche et al., 2006; Shao & Halpin, 1995)

Figure 1 shows the values of the four model evaluation measures for our two example species. Just based on how these numbers relate to the range of values that these measures can theoretically obtain (Table 1) we might either consider that both models are excellent (AUC), that the model for *C. subabruptus* is poor, whereas the model for *F. fomentarius* is relatively good (Tjur's *R* ^2^ and max‐Kappa), or that the model for *C. subabruptus* is relatively good, whereas the model for *F. fomentarius* is excellent (max‐TSS). This difference in interpretation naturally follows from the fact that the different metrics emphasize different aspects of how the predictions are in line with the data. All metrics have been advocated for some reasons and criticized for other reasons, many of which relate to how the metrics do or do not account for the overall prevalence of the species, e.g., whether they tend to generally obtain low or high values for rare species (Allouche et al., 2006; Jiménez‐Valverde et al., 2013; Lobo et al., 2008; Peterson et al., 2008; Sofaer et al., 2019). In our view, all the four measures are adequate, as long as the researcher applying them is able to correctly interpret what they do and do not measure. Here we aim to help in making such interpretations by considering an empirical case study that contains many rare species and thus where different model evaluation measures can be expected to lead to different conclusions. Earlier studies have pointed out that the spatial scale at which a model is evaluated can greatly influence predictive performance (Aguirre‐Gutiérrez et al., 2013) and that fixed and random effects play a different role in generating predictions (Roberts et al., 2017). To further illustrate how the differences between the four model evaluation metrics depend on the interplay between model structure and spatial scale of prediction, we will consider a spatially hierarchical empirical case study and set up models that vary in their random effect structures.

### 2.3 Empirical case study to examine the measures of predictive performance

As an empirical case study, we used the wood‐inhabiting fungal data of Abrego et al. (2016) surveyed at the three hierarchical spatial scales of deadwood units (i.e., sampling units), which are nested within plots, the latter being nested within sites. To simplify the case study, we selected data for 1 year only (2011) out of the originally 2‐year study. In each of the 16 forest sites, randomly located 10 × 10 m sample plots (in total 80 plots) were surveyed for the presence–absences of fruiting fungi on deadwood units of a diameter larger than 0.2 cm (in total 5097 deadwood units). We restricted the data to those 68 fungal species which were found to be present in at least 10 sampling units and for which we thus considered it possible to meaningfully fit a model and evaluate predictive performance. For each deadwood unit, the decay stage (scalar from 1 to 5) and volume (in cm^3^) were measured, and for each forest site, the management type (managed or natural) was recorded. These data allowed us to investigate the scale dependency of the discrimination measures, as well as to assess their dependency on the inclusion or exclusion of fixed and random effects.

As the SDM tool, we used the Hierarchical Modelling of Species Communities (HMSC) framework, which is a joint species distribution model (JSDM) (Ovaskainen & Abrego, 2020; Ovaskainen et al., 2017). While different SDM models can greatly vary in their absolute performance (Norberg et al., 2019), we do not expect the qualitative results of the present study to be sensitive to the specific choice of the SDM model made here, as we are not interested in absolute performance but rather understanding factors that influence performance. As the data are binary (presence or absence), we used probit regression, thus modeling occurrence probability as $\mathrm{Pr}\left(y_{\mathrm{ij}}=1\right)=\Phi \left(L_{\mathrm{ij}}\right)$, where $L_{\mathrm{ij}}$ is the linear predictor for sampling unit *i* and species *j*, and Φ is the cumulative distribution function of the standard normal distribution.

To be able to separate the roles of fixed and random effects on predictive performance, we considered three model variants: one including both fixed and random effects $\left(L_{\mathrm{ij}}=L_{\mathrm{ij}}^{F}+L_{\mathrm{ij}}^{R}\right)$, one including fixed effects only ($L_{\mathrm{ij}}=L_{\mathrm{ij}}^{F}$), and one including random effects only ($L_{\mathrm{ij}}={L_{\mathrm{ij}}^{F}+L}_{\mathrm{ij}}^{R}$, where $L_{\mathrm{ij}}^{F}$ contains only the species‐specific intercept). HMSC models the fixed effects as $L_{\mathrm{ij}}^{F}=\sum_{k}x_{\mathrm{ik}}\beta_{\mathrm{kj}}$, where $x_{\mathrm{ik}}$ is the predictor k for sampling unit *i* and $\beta_{\mathrm{kj}}$ is the response of species *j* to this predictor. We included as predictors the forest management category (a site‐level categorical covariate with two levels), the log‐transformed volume of the deadwood unit (a sampling‐unit level continuous covariate), and the decay stage of the log (a sampling‐unit level continuous covariate). We included also the second‐order term of decay stage to allow for non‐monotonic responses such as maximal occurrence probability achieved at an intermediate decay stage. HMSC models random effects through the latent‐variable approach $L_{\mathrm{ij}}^{F}=\sum_{k}\eta_{\mathrm{ik}}\lambda_{\mathrm{kj}}$, where $\eta_{\mathrm{ik}}$ is the site loading and $\lambda_{\mathrm{kj}}$ is the species loading. We included random effects at the three hierarchical levels of the study design: the site (in which case $\eta_{\mathrm{ik}}=\eta_{S\left(i\right)k}$ where $S\left(i\right)$ is the site of the sampling unit *i*), the plot (in which case $\eta_{\mathrm{ik}}=\eta_{P\left(i\right)k}$ where $P\left(i\right)$ is the plot of the sampling unit *i*), and the sampling unit. We note that while in a univariate model, a random effect at the level of the sampling unit would be fully confounded with the residual, in a multivariate JSDM its inclusion is meaningful, as it models the residual associations among the species (Ovaskainen et al., 2016). We fitted the models with the R‐package Hmsc (Tikhonov, Opedal, et al., 2020) assuming default prior distributions. For details on model fitting and evaluation of MCMC convergence, see Appendix S1.

**FIGURE 2:**
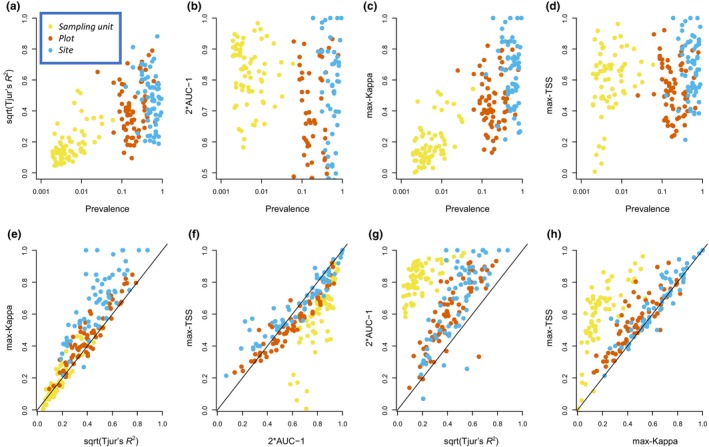
Illustration of relationships between the four measures of model performance (Tjur's *R* ^2^, AUC, max‐Kappa and max‐TSS) and prevalence (upper row of panels), and among the four measures (lower row of panels). Each dot corresponds to one species, and the results are based on the explanatory power of the full model with fixed and random effects. Each panel shows model evaluations performed at the levels of a sampling unit (yellow), plot (red), and site (blue). The lines in the lower row of panels show the identity *y* = *x*. To increase comparability among the measures, Tjur's *R* ^2^ has been square root transformed and AUC has been 2*x*−1 transformed.

We evaluated model performance separately for each species. To evaluate the scale dependency of model performance, we examined how well the models were able to predict whether the (i) sampling units (ii) plots, or (iii) sites were empty or occupied (Figure 2a). We scored a plot *P* as being occupied if at least one sampling unit belonging to it was occupied, thus defining plot‐level occurrence $y_{P}$ and computing plot‐level occurrence probability $p_{P}$ as

(1) $$y_{P}=1-\prod_{i\in P}\left(1-y_{i}\right),\;p_{P}=1-\prod_{i\in P}\left(1-p_{i}\right).$$

Similarly, we scored a site *S* as occupied if at least one sampling unit belonging to it was occupied, thus defining site‐level occurrence $y_{S}$ and computing site‐level occurrence probability $p_{S}$ as

(2) $$y_{S}=1-\prod_{i\in S}\left(1-y_{i}\right),\;p_{S}=1-\prod_{i\in S}\left(1-p_{i}\right).$$

What is critically important for the interpretation of predictive performance is how exactly the model predicted probabilities $p_{i}$ have been generated. To cover a range of cases that relate to dissimilar prediction tasks, we considered both explanatory power and three variants of predictive power. We evaluated the explanatory power by predicting the occurrence probabilities by a model fitted to all data (a procedure also known as model fit or resubstitution). We evaluated predictive power through repeated fivefold cross‐validation: splitting the sampling units into five folds (i.e., subsets), fitting the model to data on four of the folds, making predictions for the fifth fold, and looping over the five folds to generate such predictions for all sampling units. We partitioned the sampling units into the five folds following three prediction tasks in the following increasingly challenging order (Figure 2b): completely randomly (testing how well the model predicts species presence in a new sampling unit), by partitioning the plots into five folds (testing how well the model predicts species presence in a sampling unit belonging to a new plot), or by partitioning the sites into five folds (testing how well the model predicts species presence in a sampling unit belonging to a new site). To avoid dependency on the specific partitioning applied in the fivefold cross‐validation, we repeated the above procedure 10 times and averaged the results over the replicates. We selected these four model evaluation strategies (explanatory power and three variants of predictive power) as it is well known that the more independent the test units are from the training units, the worse will the predictive performance be (Bahn & McGill, 2013; Roberts et al., 2017). In particular, the random effects can be utilized for prediction only for cases where at least some sampling units have been included in the training data from the level for which the predictions are made (Ovaskainen & Abrego, 2020). By creating variation in the difficulty level of the prediction tasks, we aimed to both demonstrate its influence on the user's perception of model's performance, as well as to test whether the comparison among the four different performance measures depends on the specific prediction task. We note that even if different cross‐validation strategies are applied, our case study does not address model transferability, because it makes predictions to the same study area and to the same spatial scales (sampling units, plots, sites) to which the model was fitted.

To facilitate the comparison among the metrics, we transformed AUC as 2*AUC−1 to make all performance measure have the same range from 0 to 1 and took the square root of Tjur's *R* ^2^ due to its quadratic nature.

## 3 RESULTS

Figure 2 illustrates how the four metrics behave with respect to each other and species prevalence based on the explanatory power of the full model. Consistently with the earlier literature, AUC and max‐TSS did not have any strong relationship with prevalence whereas Tjur's *R* ^2^ and max‐Kappa generally increased with increasing prevalence (Figure 2). As prevalence increases with the spatial scale of consideration (e.g., a species is scored to be present in a plot if it is present in any of the sampling units of that plot), this resulted in Tjur's *R* ^2^ and max‐Kappa yielding much higher values for plot‐ and site‐level predictions than for sampling‐unit level predictions (Figure 2). Contrasting the four measures with respect to each other revealed that Tjur's *R* ^2^ and max‐Kappa behave largely similarly, as do AUC and max‐TSS (Figure 2). While these measures are not exactly identical, the correlations within these two pairs of measures are so high that in practice they should be seen as alternatives rather than as complementary. In contrast, a comparison between these pairs of measures shows that AUC and max‐TSS behave qualitatively differently from Tjur's *R* ^2^ and max‐Kappa (Figure 2). This is largely explained by their dependency on prevalence: all measures yield similar evaluations at the plot‐ and site‐levels where prevalence is high, but AUC and max‐TSS yield higher values than Tjur's *R* ^2^ and max‐Kappa at the sampling‐unit level where prevalence is low (Figure 2).

**FIGURE 3:**
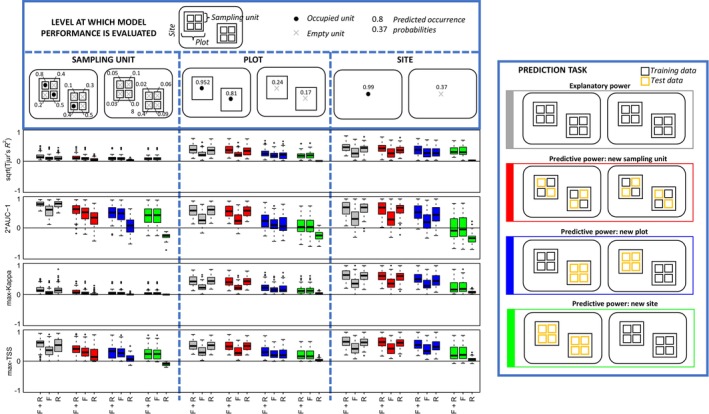
Evaluation of model performance based on the empirical case study. Each boxplot shows the distribution of model evaluation measures over the 68 species included in the study. The rows of panels correspond to Tjur's *R* ^2^ (square root transformed), AUC (2*x*−1 transformed), max‐Kappa, and max‐TSS. Model performance has been evaluated at the three hierarchical levels of sampling unit, plot, and site. The legend box on the top exemplifies how the predicted occurrence probabilities at the level of the sampling unit accumulate as occurrence probabilities at the plot and site level, respectively. The legend box on the right shows how the models have been used for four different prediction tasks that translate to different partitionings of the data into training and test sets. The results are shown for the three different model variants that contain both fixed and random effects (F & R), fixed effects only (F), or random effects only (R).

The comparison between all model types (with or without fixed and random effects) as well as all model evaluation strategies (explanatory power and predictive power based on three different model validation strategies) suggests the generality of the results reported above: Tjur's *R* ^2^ and max‐Kappa behave largely similarly, as do AUC and max‐TSS. Again, the main difference between these two pairs of measures is that Tjur's *R* ^2^ and max‐Kappa yield lower values than AUC and max‐TSS for sampling unit‐level evaluations where species prevalence is low (Figure 3). The results of Figure 3 also confirm the expectation that the more independent the test units are from the training units, the worse is the model performance (Bahn & McGill, 2013). This is especially the case for models that rely on random effects on their predictions, as the random effects can only be used for levels seen already in the training data. As one example, for models utilizing random effects, sampling unit‐level predictions are much more accurate if based on fitting the model to all data (explanatory power) rather than applying cross‐validation (predictive power) (Figure 3). As another example, for models utilizing random effects, site‐level predictions are much more accurate if based on explanatory power or predictive power computed through sampling unit‐level or plot‐level cross‐validation, as compared to predictive power computed through site‐level cross‐validation (Figure 3). These results derive from what components each model is able to use for prediction depending on the model evaluation strategy, and the fact that the random effects explained a substantial part of the explained variation also in the full model (Table S1 in the Appendix S1).

## 4 DISCUSSION

After fitting a SDM to data, a critical question to ask is “how good is the model?”. Here we showed that depending on exactly how the model's predictive performance is evaluated, the very same model can achieve very different values even for one and the same metric (be it Tjur's *R* ^2^, AUC, max‐Kappa or max‐TSS). We also showed that the four metrics of predictive performance form two groups, with Tjur's *R* ^2^ being largely similar to max‐Kappa, and AUC being largely similar to max‐TSS. These two groups of metrics behave quite differently, the main difference being that Tjur's *R* ^2^ and max‐Kappa tend to increase with prevalence whereas AUC and max‐TSS are largely independent from it. So which metrics one should use for model evaluation? As Tjur's *R* ^2^ and max‐Kappa behave similarly, as well as do AUC and max‐TSS, it seems sufficient to pick one metric from each group. Which ones to pick might mainly depend on the traditions followed in the particular research line, in order to make the results easily communicable. In our view, it is natural to choose either Tjur's *R* ^2^ and AUC, as these two measures have a probabilistic interpretation, or alternatively max‐Kappa and max‐TSS, as these two measures are derived from thresholding and as they are derived from the viewpoints of accuracy, sensitivity, and specificity.

But how do the two groups of metrics differ in terms of what exactly they measure, and do they provide complementary insights or is one of them simply a better way to assess a model's predictive performance and thus should be generally favored? To address this, let us return to the species *C. subabruptus* in Figure 1, for which the metrics are in apparent contradiction: based on the AUC value of 0.96, the model would likely to be considered excellent, whereas based on the Tjur's *R* ^2^ value of 0.02, the same model would likely be considered poor. Importantly, these two interpretations are in no contradiction, because whether the model is good or bad depends on what it is used for. For example, if a conservation biologist were to use the model to select protection units, they would consider the model not very useful, as the low Tjur's *R* ^2^ suggests that the model is unable of pinpointing the units where the species will occur with a high likelihood: even for the highest predictions, the species would actually occur only in 1 out of 10 units. However, if the same conservation biologist were to use the model to identify units that could be managed without risk of hampering the occurrences of the species, due to the high AUC they could consider the model excellent, as it successfully identifies units where the species is very unlikely to occur (say, those 90% of the sampling units for which the predicted probability is smaller than 0.005, in none of which the species is actually present). These considerations relate closely to the evaluation of predictive performance through measuring its sensitivity and specificity with respect to a particular probability threshold (Jiménez‐Valverde & Lobo, 2007; Lawson et al., 2014; Liu et al., 2011), as they relate to how important it is to correctly predict the presences (sensitivity) compared to the importance of correctly presenting the absences (specificity). Choosing a fixed threshold will, however, per necessity involve somewhat arbitrary decisions, which makes it difficult to e.g. compare predictive performances among studies (Allouche et al., 2006; Lawson et al., 2014; Liu et al., 2011; Manel et al., 2001), for which reason we have focused here on threshold independent measures.

From a statistical point of view, the value of Tjur's *R* ^2^ for *C. subabruptus* is low, because it is defined as the mean occurrence probabilities of occupied and empty sampling units, and because for this species all predictions achieved a low probability. In contrast, AUC achieved a high value, because for the vast majority of empty sampling units, the predicted occurrence probabilities are still much lower than for occupied units. Thus, following the probabilistic definition of AUC, if randomly picking one empty sampling unit and one occupied sampling unit, it is very likely that the occupied sampling unit has a higher occurrence probability. A high value of Tjur's *R* ^2^ can only be obtained when the predicted occurrence probabilities range from low (close to zero) to high (close to one), whereas a high value of AUC can be obtained even if there are only cases of either low or high predicted occurrence probabilities. These observations also relate closely to how the evaluations of model performance are influenced by the range of environmental conditions present in the sampling units. If the researcher decides to survey not only on the most suitable habitats but also include a large number of sampling units in unsuitable habitats, then the value of AUC will be inflated and theoretically converge to one if adding more and more unsuitable sampling units. While adding such sampling units will also increase the value of Tjur's *R* ^2^, the value of this metric will not converge to its maximal value of one, but to a value corresponding to the mean occurrence probability across occupied sampling units.

Our hierarchical case study demonstrated how the different model components can or cannot help in making predictions, depending on the cross‐validation strategy chosen, and the hierarchical level (or more generally, spatial scale) at which model performance is evaluated. This is in line with the earlier findings that ignoring relevant dependency structures in the cross‐validation step can greatly influence the perceived predictive performance of the model (Roberts et al., 2017). Which model evaluation strategy one should then apply, will depend on what purpose the model is to be used for. For example, if a conservation biologist uses the model to select protection sites for a threatened species, predictive performance should be evaluated at the site level and using a site‐level cross‐validation strategy. This is because the conservation biologist would like to locate occupied sites (rather than e.g., individual occupied sampling units within sites), and because for this prediction task, the researcher is unlikely to hold any species data from the candidate sites (as otherwise the model predictions would not be needed to start with). In contrast, if a manager were interested in harvesting a commercially valuable species, then a cross‐validation strategy targeting the level of sampling units might be more adequate, because that would reveal how well the model is able to identify the sampling units where the species does occur.

In this article, we have stressed that the use of simple rules of thumb for evaluating model performance can be misleading, as that the values achieved of predictive performance depend on multiple factors. This will most likely come as a disappointment for the ecologist looking for simple guidelines. How then might a researcher account for all of those confounding factors and decide on whether the model is excellent, good, fair, poor, or a complete failure? Rather than coming up with a more refined set of rules of thumb, we recommend that researchers use their intuition from past experience to derive a priori expectations on how predictable their system is, and then compare the achieved predictive performance to those expectations. By explicitly articulating this past knowledge in their expectations, they can also communicate the advances achieved by the new model. If occupied and unoccupied sites are easy to separate a priori within the data range, then what do we need a new model for? If, on the other hand, occupied and unoccupied sites are a priori close to inseparable, then even a modest improvement in our discriminatory capabilities will correspond to a major advancement.

For example, based on our field experience on surveying wood‐inhabiting fungi, we know that *C. subabruptus* is not particularly affected by forest management, that it is absent from the smallest deadwood pieces, and that it appears to slightly prefer intermediate decay classes. Given all this knowledge, our intuition tells that it is possible to predict beforehand that the species will be absent from certain kinds of deadwood units, but it will be very difficult to tell exactly in which deadwood units this species is present, which is in line with the low value of Tjur's *R* ^2^ and high value of AUC achieved at the level of deadwood units. For the other example species of Figure 1, *F. fomentarius*, our field intuition tells that the species is quite predictably found from large deadwood units and absent from small ones, which is in line with the high values of Tjur's *R* ^2^ and AUC that we obtained for this species. These kinds of considerations can be generalized to the levels of study systems and spatial scales. For example, while for an ecologist conducting a field survey it can be very difficult to predict which individual deadwood units will be occupied by a given fungal species, it can be much easier to predict in which forest sites that species will occur, as was observed by the higher values of Tjur's *R* ^2^ that we recorded at the plot and site levels. As another example, for a researcher surveying birds in forest fragments, it can be difficult to predict beforehand in which sites the common chaffinch (*Fringilla coelebs*) will or will not be found, because the species is a forest generalist. However, if the survey was extended to also cover many other types of habitats, such as cultivated areas and water bodies, then it will be much easier to beforehand predict that the chaffinch will mostly be found in the forest sites.

One reason why Tjur's *R* ^2^ may have not gained much popularity in ecological studies is that it generally yields much lower values than AUC. The “modest” values achieved can then be harder to communicate and publish than the typically much higher AUC values. As a reviewer, one may be misled to condemn a model as inferior as based on a low Tjur's *R* ^2^, unless one gives added thought to why this is the case. Given the complementary information contained in the two measures, and the fact that most ecologists are typically more familiar with the scale of AUC, our recommendation is for researchers to report both measures (or alternatively, max‐Kappa and max‐TSS) and to spell out their assessment of how these values relate to a priori expectations. Only this way may the author and the reader evaluate the values from the same starting positions. With this article, we hope we have increased the general awareness about the options for evaluating predictive performance, the complications inherent and their implications for the values achieved—thus allowing researchers to reach more comprehensive and informative assessments on how useful their models are for the very purposes that these models were built.

## AUTHOR CONTRIBUTIONS

**Nerea Abrego:** Conceptualization (equal); formal analysis (equal); investigation (equal); methodology (equal); resources (equal); writing – original draft (equal); writing – review and editing (equal). **Otso Ovaskainen:** Conceptualization (equal); formal analysis (equal); investigation (equal); methodology (equal); resources (equal); writing – original draft (equal); writing – review and editing (equal).

## CONFLICT OF INTEREST STATEMENT

The authors declare no conflict of interest.

## Supporting information


Appendix S1.

## Data Availability

The data and scripts to reproduce the results of this study have been published in Zenodo (https://zenodo.org/record/8281224).
