# Supplementary material for: Evaluating the predictive performance of presence–absence models: Why can the same model appear excellent or poor?
Source: Ecol Evol. 2023 Dec 18;13(12):e10784. doi: 10.1002/ece3.10784 (PMC10726276; doi:10.1002/ece3.10784)
Supplement: Supplementary file 1 — Appendix S1. [file ECE3-13-e10784-s001.docx]

# **Supporting information for:**

Evaluating the predictive performance of presence-absence models: why can the same model appear excellent or poor?

### **Details on the HMSC model: model fitting, MCMC convergence, and variance partitioining**

We fitted the HMSC model with the R-package Hmsc (Tikhonov et al., 2020) assuming the default prior distributions (Ovaskainen & Abrego, 2020, Chapter 8). We sampled the posterior distribution with four Markov Chain Monte Carlo (MCMC) chains, each of which was run for 37,500 iterations, of which the first 12,500 were removed as burn-in. The chains were thinned by 100 to yield 250 posterior samples per chain, thus 1000 posterior samples in total. We examined MCMC convergence for the full model with fixed and random effects (which is the most complex model and hence the most difficult for achieving MCMC convergence), by examining the potential scale reduction factors (Gelman & Rubin, 1992) of the model parameters that measure fixed effects and random effects. The MCMC convergence was satisfactory:

- For the $\beta$ parameters that relate to the fixed effects, the mean (3^rd^ quartile, max) potential scale reduction factor was 1.01 (1.01, 1.20).
- For the $\Omega$ parameters that relate to the random effects at the site-level, the mean (3^rd^ quartile, max) potential scale reduction factor was 1.01 (1.02, 1.26).
- For the $\Omega$ parameters that relate to the random effects at the plot-level, the mean (3^rd^ quartile, max) potential scale reduction factor was 1.01 (1.01, 1.13).
- For the $\Omega$ parameters that relate to the random effects at the sampling unit -level, the mean (3^rd^ quartile, max) potential scale reduction factor was 1.03 (1.04, 1.39).

**Table S1.** Variance partitioning among fixed and random effects included in the models. The values show the average (over species) proportions of explained variance attributed to each component of the model. Fixed effects at the level of the sampling unit include the volume of the deadwood unit and the second order polynomial of decay stage, whereas fixed effects at the site level include management category. Note that the models did not include any fixed effects at the plot level, for which reason they explain no variation at this level.

| Model variant  Model component | Fixed and random | Fixed only | Random only |
| --- | --- | --- | --- |
| Fixed: sampling unit | 0.51 | 0.85 | NA |
| Fixed: plot | NA | NA | NA |
| Fixed: site | 0.08 | 0.15 | NA |
| Random: sampling unit | 0.11 | NA | 0.44 |
| Random: plot | 0.15 | NA | 0.27 |
| Random: site | 0.15 | NA | 0.29 |

**References**

Gelman, A., & Rubin, D. B. (1992). Inference from iterative simulation using multiple sequences. *Statistical Science*, *7*(4). https://doi.org/10.1214/ss/1177011136

Ovaskainen, O., & Abrego, N. (2020). *Joint Species Distribution Modelling: With Applications in R* (1st ed.). Cambridge University Press. https://doi.org/10.1017/9781108591720

Tikhonov, G., Opedal, Ø. H., Abrego, N., Lehikoinen, A., Jonge, M. M. J., Oksanen, J., & Ovaskainen, O. (2020). Joint species distribution modelling with the R-package Hmsc. *Methods in Ecology and Evolution*, *11*(3), 442–447. https://doi.org/10.1111/2041-210X.13345
